# Effectiveness of chitosan-propolis nanoparticle against *Enterococcus faecalis* biofilms in the root canal

**DOI:** 10.1186/s12903-020-01330-0

**Published:** 2020-11-25

**Authors:** Abhishek Parolia, Haresh Kumar, Srinivasan Ramamurthy, Fabian Davamani, Allan Pau

**Affiliations:** 1 Division of Clinical Dentistry, School of Dentistry, International Medical University, Kuala Lumpur, Malaysia; 2 Department of Pathology, School of Medicine, International Medical University, Kuala Lumpur, Malaysia; 3 College of Pharmacy and Health Sciences, University of Science and Technology of Fujairah, Fujairah, UAE; 4 School of Health Sciences, International Medical University, Kuala Lumpur, Malaysia

**Keywords:** Dentinal tubule disinfection, *Enterococcus faecalis*, Intracanal medicaments, Chitosan-propolis nanoparticle

## Abstract

**Background:**

The successful outcome of endodontic treatment depends on controlling the intra-radicular microbial biofilm by effective instrumentation and disinfection using various irrigants and intracanal medicaments. Instrumentation alone cannot effectively debride the root canals specially due to the complex morphology of the root canal system. A number of antibiotics and surfactants are being widely used in the treatment of biofilms however, the current trend is towards identification of natural products in disinfection. The aim of the study was to determine the antibacterial effect of chitosan-propolis nanoparticle (CPN) as an intracanal medicament against *Enterococcus faecalis* biofilm in root canal.

**Methods:**

240 extracted human teeth were sectioned to obtain 6 mm of the middle third of the root. The root canal was enlarged to an internal diameter of 0.9 mm. The specimens were inoculated with *E. faecalis* for 21 days. Following this, specimens were randomly divided into eight groups (*n* = *30*) according to the intracanal medicament placed: group I: saline, group II: chitosan, group III: propolis100 µg/ml (P100), group IV: propolis 250 µg/ml (P250), group V: chitosan-propolis nanoparticle 100 µg/ml (CPN100), group VI: chitosan-propolis nanoparticle 250 µg/ml (CPN250), group VII: calcium hydroxide(CH) and group VIII: 2% chlorhexidine (CHX) gel. Dentine shavings were collected at 200 and 400 μm depths, and total numbers of CFUs were determined at the end of day one, three and seven. The non-parametric Kruskal Wallis and Mann–Whitney tests were used to compare the differences in reduction of CFUs between all groups and probability values of p < 0.05 were set as the reference for statistically significant results. The scanning electron microscope (SEM) and confocal laser scanning microscopy (CLSM) were also performed after exposure to CPNs. The effectiveness of CPNs were also evaluated against *E. faecalis* isolated obtained from patients having failed root canal treatment.

**Results:**

The treatments of chitosan, P100, P250, CPN100, CPN250, CH and 2% CHX reduced the CFUs significantly compared to saline (p < .05). On day one and three, at 200 and 400-μm, CPN250 showed significant reduction of CFUs compared to all other groups (p < .05), while CPN100 was significantly better than other groups (p < .05) except CPN250 and 2% CHX. On day seven, at 200-μm CPN250 showed significant reduction of CFUs compared to all other groups (p < .05) except CPN100 and CHX, while at 400 μm CPN250 showed similar effectiveness as CPN100, CH and 2% CHX. SEM images showed root canal dentin treated with CPN250 had less coverage with *E. faecalis* bacteria similarly, CLSM images also showed higher percentage of dead *E. faecalis* bacteria with CPN250 than to CPN100.

**Conclusion:**

CPN250 was the most effective in reducing *E. faecalis* colonies on day one, three at both depths and at day seven CPN250 was equally effective as CPN100 and 2% CHX.

## Background

The primary objectives of root canal therapy are to remove infection and prevent reinfection in the root canal system [1]. Persistence of micro-organisms inside the root canal system is the most common reason for the failure of root canal therapy [2]. Microbiota in the root canal system are found in highly organized and complex entities known as biofilms [3, 4]. The complexity, variability of root canal system along with the nature of biofilm makes the root canal disinfection extremely challenging [5, 6]. Bacteria in the biofilms are particularly resistant to treatment due to their resistance to penetration by anti-microbials and express more virulent phenotypes when growing inside biofilms than as planktonic forms [4]. Within a biofilm, a wide variety of bacteria are found forming a multi-species community however, *E. faecalis* has been found most frequently in persistent intraradicular infections [7–9]. It is a gram-positive, facultative anaerobic bacterium that can survive in harsh conditions due to its ability to create biofilm, compete with other microorganisms, invade dentinal tubules, and resist nutritional deprivation [10–15]. Therefore, the successful outcome of endodontic treatment depends on controlling the intra-radicular microbial biofilm by effective instrumentation and disinfection using various irrigants/medicaments.

Instrumentation alone cannot effectively debride the root canals specially due to the complex morphology of the root canal system [16], moreover, bacteria can penetrate deep into dentinal tubules upto 1500 µm of the root canal [17–19]. Conventional root canal formulations like gel, solution and other form of intracanal medicaments are inaccessible to bacteria because they have limited penetrability into the dentinal tubules [20]. Though, a number of antibiotics and surfactants are being widely used in the treatment of biofilms however, the current trend is towards identification of natural products in disinfection.

The flavonoids from propolis ethanolic extracts are proven to have antibacterial property [21]. Bees use propolis to reinforce their hive walls and protect the hives from infection. It is a green–brown, brown or black colour resinous, balsamic substance with sharp bitter flavour and a sweet, agreeable aroma. It is composed of resin, balsams, essential oils, flavonoids, phenols, aromatic compounds, wax, pollen, amino acids, vitamins and minerals [22]. Similarly, chitosan a cationic biopolymer has been of a great interest in the recent past mainly due to its low toxicity and bio-adhesive properties. It’s positive charge allows the complex formation with oppositely charged molecules, interacting readily with negatively charged compounds. Such complexes may be used as delivery systems for incorporating a number of bioactive compounds to reduce biofilm bacteria [23, 24].

Along with the disinfecting action, the size of nanoparticles plays an important role in the antibacterial activity. Studies have reported that smaller size particles show higher antibacterial activity than the macro scaled ones [25, 26]. Since most nanoparticles used in for treating biofilm mediated infections contain metals or drugs [27] nanoformulations with natural products may provide broader potential for therapy. Therefore, the aim of this study was to evaluate the antibacterial effect of such products including chitosan-propolis nanoparticle (CPN) against *E. faecalis* biofilm in root canal dentinal tubules at depths of 200 and 400 µm and compare with routinely used intracanal medicaments such as calcium hydroxide (CH) and 2% chlorhexidine (CHX).

## Methods

This study was approved by IMU Joint-Committee on Research and Ethics under the research project ERGS/1/2013/SKK11/IMU/03/01.

The effectiveness of CPN as an intracanal medicament was evaluated against the strain *E. faecalis *(*ATCC 29212*) in human tooth model. The effectiveness of CPN was also assessed using SEM and CLSM. Another experiment was carried out to evaluate the effectiveness of CPN as an intracanal medicament against *E. faecalis* isolates from patients with failed root canal treatment.

### Preparation of ethanolic extracts of Malaysian propolis

Malaysian propolis was collected from bee farm, Pahang, Malaysia with the following geographical coordinates: north latitude 3.8126°, east latitude 103.3256° and height of 12 m above sea level. There was no permission required to collect Propolis.

The extraction method used in this study was similar to the method explained by Jacob et al*.* [28]. Propolis was manually cut into small pieces, 40 g were weighed using a weighing balance (Pyrometro, Malaysia) and divided equally into four pieces of ten grams each. Just after, in a flask, 20% (w/v) extract of propolis was prepared using 80% ethanol under constant agitation in a rotary shaker (Certomat Model S II, Sartorius, Goettingen, Germany) at 200 rpm, 37 °C for 48 h. This was later centrifuged (Eppendorf Model 5810 R, Hamburg, Germany) at 3000 rpm for 15 min, filtered through Whatman no.1 filter paper and subjected to reduced pressure using a rotary evaporator (Buchi Rotavapor R-215, Flawil, Switzerland) at the set pressure 175 mBar, temperature 52 °C and speed 95 rpm to remove the solvent. The ethanolic extract of propolis was then stored in a glass container and left for three days to allow evaporation of the residual solvent resulting in extracts of propolis (final weight/initial weight × 100). Stock solutions of 1 mg/ml of the extracts were prepared to use in further experiments. Saline with 0.1% DMSO was used to prepare the stock solution of propolis. To study the content of Malaysian propolis, reversed phase high performance liquid chromatography (RP-HPLC) analysis was carried out. The flavonoids such as pinocembrin (5.90 µg/ml), kaempferol (5.88 µg/ml) and quercetin (1.43 µg/ml) were identified to be in the highest concentration in Malaysian propolis [29].

### Preparation and characterization of CPN

CPN were prepared by ionotropic gelation of chitosan with sodium TPP according to the method reported by Koukaras et al. [30]. Stock solutions of 0.2% w/v of chitosan and 0.15% w/v sodium TPP were prepared by mixing in 1% v/v acetic acid and distilled water, respectively. The pH of both solutions was adjusted to between pH 5.0 and 5.5 by adding acetic acid. The different concentrations of ethanol extract of propolis was dissolved in chitosan solution with continuous stirring. The chitosan solutions containing propolis was added into the TPP solution and continuously stirred at 400–600 rpm at 37 °C. The nanoparticles were formed spontaneously due to ionic interaction. Following this, the formed nanoparticles were separated by centrifugation at 11,000 rpm for 25 min and the supernatants were discarded. CPN were resuspended in purified water for further characterization.

In this study, CPN (0.2% w/v chitosan and 1 mg/ml propolis) was used with an average particle size of 107.74 ± 0.53 nm, zeta potential of 45.2, polydispersity index of 0.225, and encapsulation efficiency of 88.8%. The shape of nanoparticles was observed using transmission electron microscopy. It was spherical in shape with a smooth surface similar to study done by Ong et al. [29].

### Antibacterial effect of CPN as an intracanal medicament against *E. faecalis* (ATCC 29,212) in human tooth model

#### Preparation of dentine block specimens

In this study, the experiments were carried out in extracted human tooth model, a modification of Haapasalo & Orstavik tooth model in which bovine teeth were used. This provided a better simulation to clinical settings to assess the antibacterial effectiveness of intracanal medicaments in the dentinal tubules. This protocol is similar to study done by Chua et al*.* [31]

A total of 240 sound human teeth, including maxillary anterior teeth and mandibular canines with complete root formation were included in this study. The teeth were cleaned and stored in saline during all procedures to avoid dehydration. A low-speed diamond disc (Bredent^®^, Wittighausen, Senden, Germany) mounted on a milling machine under water cooling was used to section the teeth between cementoenamel junction and the apical third of the root to obtain 6 mm of the middle third of the root. Peeso Reamer no. 2 (Mani^®^, Utsunoniya, Tochigi, Japan) in a low-speed hand piece (Kavo, Charlotte, North Carolina, USA) was used to standardise the internal diameter of root canals to 0.9 mm. The dentine blocks were subjected to sonic irrigation (EndoActivator, Dentsply, Weybridge, Surrey, UK) using 5.25% NaOCl (Clorox^®^, Oakland, California, USA) and then 17% EDTA (Calasept^®^, Nordiska Dental, Ängelholm, Skåne Country, Sweden) for two minutes to remove smear layer. The dentine block specimens were thoroughly rinsed with sterile saline after each irrigation. Following this, the dentine blocks were sterilised by autoclave (LTE^®^, Oldham, Lancashire, UK) at 121 °C for 20 min. In order to prevent any contact of *E. faecalis* and medicament with the external surface, nail varnish was applied to the outer surface of the specimen. Petri dishes containing wax with a flat surface were prepared, and surface was disinfected using 70% ethanol and later air dried in a sterile biosafety cabinet before use. All experiments were done in the laminar hood after the ultraviolet sterilization. The dentine block specimens were placed upright with the apical ends fixed to the petri dishes with wax, using a thin small square of sterilised parafilm (Parafilm M^®^, Brand, Wertheim, Baden-Württemberg, Germany) obliterating the apical orifice to prevent any softened wax from entering the root canals.

#### E. faecalis inoculation

*E. faecalis (ATCC 29212)* were suspended in 20.0 ml of tryptic soy broth (TSB) (BD DifcoTM, NJ, USA). The cell suspension was adjusted to match the turbidity of 1.5 × 10^8^ CFUs /ml (equivalent to 0.5 McFarland standards). The *E. faecalis* inoculum were transferred into the dentine block specimens using sterile 5.0 ml syringe (Terumo^®^, Somerset, New Jersey, USA) with 30-gauge needles (Terumo, Somerset, New Jersey, USA) in a sterile laminar flow hood. The coronal part of the dentine blocks was then sealed immediately using parafilm (Parafilm M^®^, Brand, Wertheim, Baden-Württemberg, Germany). Following this inoculation, the dentine block specimens were incubated for 21 days at 37 °C. The root canals were replenished with *E. faecalis* inoculum every three days to supply nutrients to bacteria and prevent their death.

#### Intracanal medicament placement

Following the inoculation period, 240 dentine blocks were randomly divided into eight groups (n = 30) according to the intracanal medicament placed: group I: saline, group II: chitosan, group III: propolis100 µg/ml (P100), group IV: propolis 250 µg/ml (P250), group V: chitosan-propolis nanoparticle100 µg/ml (CPN100), group VI: chitosan-propolis nanoparticle 250 µg/ml (CPN250), group VII: calcium hydroxide (CH) and group VIII: 2% chlorhexidine gel (2% CHX) (Consepsis V^®^, Ultradent, UT, USA).

Each group was further divided into three subgroups based on the time period (day one, three and seven) of the intracanal medicament placed. The intracanal medicaments were placed in the canal using a sterile 5.0 ml syringes (Terumo^®^, NJ, USA) and gel etchant needle tip (Kerr^®^, CA, USA) until the canals were completely filled. Thereafter, the coronal orifices were sealed using Parafilm (Parafilm M^®^, Brand, Wertheim, Germany). The blocks were kept in incubator at 37 °C for the experimental period of one, three and seven days.

#### Dentinal shavings collection

At the end of one, three and seven days, the dentine blocks were removed from the petri dishes and the canals were dried with sterile paper points. Samples of dentinal shavings were collected from all groups after one day of exposure, after three days of exposure and after seven days of exposure. Dentinal shavings were collected using peeso reamer (Mani^®^, Utsunoniya, Tochigi, Japan) size no. 4 equivalent to 1.3 mm diameter followed by size no. 6 equivalent to 1.7 mm diameter using a low speed handpiece (Kavo^®^, Charlotte, North Carolina, USA). Only one stroke was made to standardize the volume of dentinal debris collected.

#### Antimicrobial assessment

The collected dentinal shavings were transferred into a micro-centrifuge tube (Axygen, NY, USA) containing 1 ml sterile tryptic soy broth (TSB) (BD DifcoTM, NJ, USA). A sterile microtip was used to take 100 µl of broth containing dentinal shavings and transferred to another tube containing 900 µl sterile tryptic soy broth (TSB) (BD DifcoTM, NJ, USA). The content of each tube was then serially diluted from 10^–1^ until 10^–4^. Subsequently, 300 µl of the diluted dentinal shavings was streaked uniformly using a L-shaped glass rod and triplicated. These tryptic soy agar plates (BD DifcoTM, NJ, USA) were incubated at 37 °C for 24 h. Following the incubation, the colonies were counted, and readings were tabulated.

Total numbers of CFUs were calculated to determine the remaining viable microbial population. The SPSS computer software version 21.0 (SPSS Inc., Chicago, Illinois, USA) was used to perform statistical analysis. Mean CFUs were compared between the groups and subgroups. Additionally, mean difference in CFUs between the groups based on different time periods and dentinal tubules depths was compared.

The data distribution was assessed for normality and was found that it did not follow a normal distribution. Therefore, non-parametric tests including Kruskal–Wallis test and Mann Whitney U test were used to compare CFUs between the groups and subgroups of intracanal medicaments and endodontic irrigants at different time periods and depths of dentinal tubules. Probability values of p < 0.05 were set as the reference for statistically significant results.

### SEM analysis

Dentinal blocks (n = 3 per group) were prepared using the same method as mentioned above under the dentine block specimens for SEM analysis before and after treatment. *E. faecalis (ATCC 29,212)* was cultured in 10 ml TSB (BD DifcoTM, NJ, USA) added with 8% sucrose with pH 7.4 and a minimal amount of xylitol (0–2%) at 37 °C for 48 h. This broth was incubated at 37 °C for 24 h. After centrifugation using 4000 rpm for 15 min, each cell pellet was washed thrice with sterile phosphate buffered solution (0.01 M, pH 7.2). Thereafter, it was re-suspended in 10 ml of the growth medium to adjust its concentration similar to 0.5 McFarland units (10^8^ cells/ml) before use. The bacterial inoculum was mixed in five millilitres of TSB (BD DifcoTM, NJ, USA) and transferred into to root canal using sterilised syringe for a period of 21 days. The bacterial inoculation was similar to the method previously described in human tooth model used in this study. After 21 days, intracanal medicaments were placed according to the groups mentioned above. Two parallel grooves were created using a diamond disc onto the external surfaces of the dentin specimen in mesio-distal direction to facilitate a split fracture. Final splitting was done using chisel and hammer. Following this, all specimens were dehydrated in ascending grades of ethanol for 20 min each and immediately transferred into the pressure chamber of the critical point drying machine (CPD 30; Leica). All specimens were mounted on aluminium stubs using double-sided conductive tape and 30 nm-thick layer gold sputtering was done for two minutes. Following this, the specimens were examined using SEM (Philips/FEI XL30 FEG SEM, Japan) at an accelerated voltage of 5 kV at different magnifications and images were evaluated. Different magnifications and images were observed to evaluate the qualitative reduction of *E. faecalis*. Four-score scale system based on percentage of residual isolated microbial cells was used to assess the microbial coverage on SEM images of the canal walls [32]. The scores were defined as clean dentine or residual isolated microbial cells covering less than 5% of the dentine, covering 5%—33% of the dentine, 34%—66% of dentine and 67%—100% of the dentine.

### CLSM analysis

This analysis was conducted to evaluate the effectiveness of CPN250 and CPN100 as intracanal medicaments by assessing the viability profile. The proportion of live and dead bacteria was determined by fluorescent staining followed by imaging. The protocol used in this study was similar to done by Dawood et al*.* [33]

After the disinfection solution regimen, the specimen (n = 1 in each group) was rinsed in 0.1% by weight fluorescein for 24 h. Specimen were thereafter rinsed with deionised water and examined using CLSM (Leica Fluoview FV 1000, Olympus, Tokyo, Japan) equipped with a 60 × /1.4 NA oil immersion lens using 488 nm argon/helium and a 633 nm krypton ion laser illumination in reflection as well as fluorescence modes. Reflected and fluorescence signals were detected using a photomultiplier tube to a depth of 20 μm and then converted to single-projection images for better visualisation and qualitative analysis. Stacks of fluorescent images of the biofilm were obtained and examined using BioimageL software (v.2.0. Malmő, Sweden). This software provides information on the structure of the biofilm, including green-stained indicating live bacteria and red-stained indicating dead bacteria and volume on a two-dimensional x–y section based on colour segmentation algorithms written in MATLAB.

### Antibacterial effect of CPN as an intracanal medicament against *E. faecalis* isolates from clinical samples

#### Patient selection

Ten patients aged between 20 and 60 years were selected from those who attended the IMU Oral Health Centre, Kuala Lumpur, Malaysia, needing endodontic retreatment. A detailed medical and dental history were obtained from each patient. Patients who have systemic disease or have received antibiotic treatment during the last three months were excluded from the study to minimise any risk of bias. Ten teeth from ten different patients with failed root canal treatment were included in this experiment. Failure of root-canal treatment was determined on the basis of clinical examination such as presence of pain, tenderness, swelling, sinus opening and mobility and radiographical examinations such as persistence of periapical lesion and root resorption.

#### Sampling procedure

After explaining the complete process of investigation including the method of sample collection, a written informed consent was obtained. Thereafter, the retreatment procedure was carried out. An access cavity was prepared under syringe irrigation using sterile high-speed diamond bur. Root-filling material was removed by rotary instrumentation and K-files (Dentsply-Maillefer, Ballaigues, Switzerland) in a crown-down technique without the use of chemical solvent, accomplished by irrigation with sterile saline. Following this, a sterile paper point (Dentsply-Maillefer, Ballaigues, Switzerland) was then introduced into the full length of the canal and retained in position for one minute for sampling. Culture procedure was done using the selective *E. faecalis* plates (Slanetz Bartley Agar (m-Enterococcus A.), Liofilchem, Italy) and the bacteria were grown and identified.

To prepare the *E. faecalis* inoculum, these isolates were suspended in 20.0 ml of TSB (BD DifcoTM, NJ, USA) and adjusted to match the turbidity of 1.5 × 10^8^ CFUs /ml (equivalent to 0.5 McFarland standards) similar to the method describe above. Thereafter, one ml of this *E. faecalis* suspension was transferred into the Eppendorf tube containing 50 µl of each medicament according to these eight groups Group I: Saline, Group II: Chitosan, Group III: P100, Group IV: P250, Group V: CPN100, Group VI: CPN250, Group VII: CH and Group VIII: 2% CHX and incubated. After day one, three and seven, the content of each tube was serially diluted as described above in this study. 300 µl of the diluted shavings was streaked evenly using a L-shaped glass rod and triplicated. Thereafter, these plates were incubated at 37 °C for 24 h, bacteria were grown CFUs were calculated.

## Results

The accuracy of the methodology was validated by observing the large amount of *E. faecalis* CFUs in the saline (control group) at all experimental timings.

On statistical analysis, the mean reduction in CFUs was found to be significant (p < 0.05) in all the groups when compared to the saline group at all times and all depths. On day one and day three, at 200 and 400 μm depths of the dentinal tubules, CPN250 showed significant mean reduction of CFUs (p < 0.05) when compared to all other groups. Furthermore, on day one and day three, at 200 and 400 μm, CPN100 showed statistically significant mean reduction of CFUs (p < 0.05) when compared to other groups except CPN250 and 2% CHX. On day seven, at 200 μm depth, CPN250 showed statistically significant mean reduction of CFUs when compared to all other groups (p < 0.05) except CPN100 and 2% CHX while at 400 μm, no significant difference was observed in between CPN250, CPN100, CH and 2% CHX (Table 1).

**Table 1:** Mean difference in CFUs between the groups on day one, three and seven at 200 and 400 µm depth

Mean difference in CFUs between the groups	Mean difference in CFUs day one at 200 µm depth	p value	Mean difference in CFUs day one at 400 µm depth	p value	Mean difference in CFUs day three at 200 µm depth	p value	Mean difference in CFUs day three at 400 µm depth	p value	Mean difference in CFUs day seven at 200 µm depth	p value	Mean difference in CFUs day seven at 400 µm depth	P value
Saline versus Chitosan	2.52 × 10^6^*	0.000	2.23 × 10^6^*	0.000	3.11 × 10^6^*	0.000	2.56 × 10^6^*	0.000	3.14 × 10^6^*	0.000	3.10 × 10^6^*	0.000
Saline versus P100	3.31 × 10^6^*	0.000	3.07 × 10^6^*	0.000	3.41 × 10^6^*	0.000	3.06 × 10^6^*	0.000	3.23 × 10^6^*	0.000	3.19 × 10^6^*	0.000
Saline versus P250	3.33 × 10^6^*	0.000	3.08 × 10^6^*	0.000	3.42 × 10^6^*	0.000	3.07 × 10^6^*	0.000	3.23 × 10^6^*	0.000	3.19 × 10^6^*	0.000
Saline versus CPN100	3.44 × 10^6^*	0.000	3.16 × 10^6^*	0.000	3.49 × 10^6^*	0.000	3.12 × 10^6^*	0.000	3.24 × 10^6^*	0.000	3.19 × 10^6^*	0.000
Saline versus CPN250	3.53 × 10^6^*	0.000	3.29 × 10^6^*	0.000	3.53 × 10^6^*	0.000	3.18 × 10^6^*	0.000	3.24 × 10^6^*	0.000	3.19 × 10^6^*	0.000
Saline versus CH	3.20 × 10^6^*	0.000	3.09 × 10^6^*	0.000	3.41 × 10^6^*	0.000	3.05 × 10^6^*	0.000	3.23 × 10^6^*	0.000	3.19 × 10^6^*	0.000
Saline versus 2% CHX	3.48 × 10^6^*	0.000	3.19 × 10^6^*	0.000	3.52 × 10^6^*	0.000	3.15 × 10^6^*	0.000	3.23 × 10^6^*	0.000	3.19 × 10^6^*	0.000
Chitosan versus P100	7.92 × 10^5^*	0.000	8.45 × 10^5^*	0.000	2.90 × 10^5^*	0.000	5.02 × 10^5^*	0.000	9.33 × 10^4^*	0.000	8.93 × 10^4^*	0.000
Chitosan versus P250	8.10 × 10^5^*	0.000	8.56 × 10^5^*	0.000	3.05 × 10^5^*	0.000	5.04 × 10^5^*	0.000	9.35 × 10^4^*	0.000	8.93 × 10^4^*	0.000
Chitosan versus CPN100	9.13 × 10^5^*	0.000	9.26 × 10^5^*	0.000	3.71 × 10^5^*	0.000	5.59 × 10^5^*	0.000	9.38 × 10^4^*	0.000	8.97 × 10^4^*	0.000
Chitosan versus CPN250	1.00 × 10^6^*	0.000	1.06 × 10^6^*	0.000	4.15 × 10^5^*	0.000	6.18 × 10^5^*	0.000	9.38 × 10^4^*	0.000	8.96 × 10^4^*	0.000
Chitosan versus CH	6.76 × 10^5^*	0.001	8.60 × 10^5^*	0.000	2.97 × 10^5^*	0.000	4.85 × 10^5^*	0.000	9.36 × 10^4^*	0.000	8.95 × 10^4^*	0.000
Chitosan versus 2% CHX	9.54 × 10^5^*	0.001	9.59 × 10^5^*	0.000	4.02 × 10^5^*	0.000	5.90 × 10^5^*	0.000	9.38 × 10^4^*	0.000	8.96 × 10^4^*	0.000
P100 versus P250	1.77 × 10^4^	0.199	1.00 × 10^4^	0.545	1.50 × 10^4^	0.70	2.46 × 10^3^	0.677	1,32 × 10^2^	0.129	60	0.590
P100 versus CPN100	1.21 × 10^5^*	0.000	8.06 × 10^4^*	0.001	8.10 × 10^4^*	0.000	5.72 × 10^4^*	0.000	4.42 × 10^2^*	0.001	3.95 × 10^2^*	0.001
P100 versus CPN250	2.15 × 10^5^*	0.000	2.17 × 10^5^*	0.000	1.24 × 10^5^*	0.000	1.16 × 10^5^*	0.000	4.42 × 10^2^*	0.001	3.12 × 10^2^*	0.008
P100 versus CH	–1.15 × 10^5^	0.650	1.41 × 10^4^	0.450	6.36 × 10^3^	0.496	–1.68 × 10^4^	0.290	2.55 × 10^2^*	0.023	2.00 × 10^2^	0.134
P100 versus 2% CHX	1.61 × 10^5^*	0.000	1.13 × 10^5^*	0.000	1.11 × 10^5^*	0.000	8.83 × 10^4^*	0.000	4.40 × 10^2^*	0.001	3.40 × 102*	0.003
P250 versus CPN100	1.03 × 10^5^*	0.000	7.05 × 10^4^*	0.001	6.60 × 10^4^*	0.000	5.47 × 10^4^*	0.000	310*	0.001	3.35 × 10^2^*	0.002
P250 versus CPN250	1.97 × 10^5^*	0.000	2.07 × 10^5^*	0.000	1.09 × 10^5^*	0.000	1.14 × 10^5^*	0.000	3.10 × 10^2^*	0.001	2.52 × 10^2^*	0.027
P250 versus CH	–1.33 × 10^5^	0.940	4.02 × 10^3^	0.821	–8.67 × 10^4^	0.496	–1.92 × 10^4^	0.226	1.22 × 10^2^	0.173	1.40 × 10^2^	0.277
P250 versus 2% CHX	1.43 × 10^5^*	0.000	1.03 × 10^5^*	0.000	9.63 × 10^4^*	0.000	8.59 × 10^4^*	0.000	3.07 × 10^2^*	0.001	2.80 × 10^2^*	0.012
CPN100 versus CPN250	9.42 × 10^4^*	0.000	1.37 × 10^5^*	0.000	4.35 × 10^4^*	0.000	5.92 × 10^4^*	0.000	0	1.00	–82.5	0.147
CPN100 versus CH	–2.37 × 10^5^*	0.000	–6.65 × 10^4^*	0.005	–7.46 × 10^4^*	0.000	–7.40 × 10^4^*	0.000	− 1.87 × 10^2^*	0.005	− 1.95 × 10^2^*	0.030
CPN100 versus 2% CHX	4.02 × 10^4^*	0.002	3.28 × 10^4^	0.070	3.03 × 10^4^*	0.000	3.11 × 10^4^*	0.005	− 2.5	0.317	− 55	0.146
CPN250 versus CH	− 3.31 × 10^5^*	0.000	− 2.03 × 10^5^*	0.000	− 1.18* × 10^5^	0.000	− 1.33 × 10^5^*	0.000	− 1.87 × 10^2^*	0.005	− 1.12 × 10^2^	0.281
CPN250 versus 2% CHX	− 5.39 × 10^4^*	0.000	− 1.04 × 10^5^*	0.000	− 1.31 × 10^4^*	0.000	− 2.80 × 10^4^*	0.000	− 2.5	0.317	27.5	0.914
CH versus 2% CHX	2.77 × 10^5^*	0.000	9.94 × 10^4^*	0.000	1.05 × 10^5^*	0.000	1.05 × 10^5^*	0.000	1.85 × 10^2^*	0.013	1.40 × 10^2^	0.191

*Statistically significant (p < 0.05), the number denotes mean difference of CFUs between the group in the row (-value denotes the first group in the row is better than the second)

**Fig. 1:**
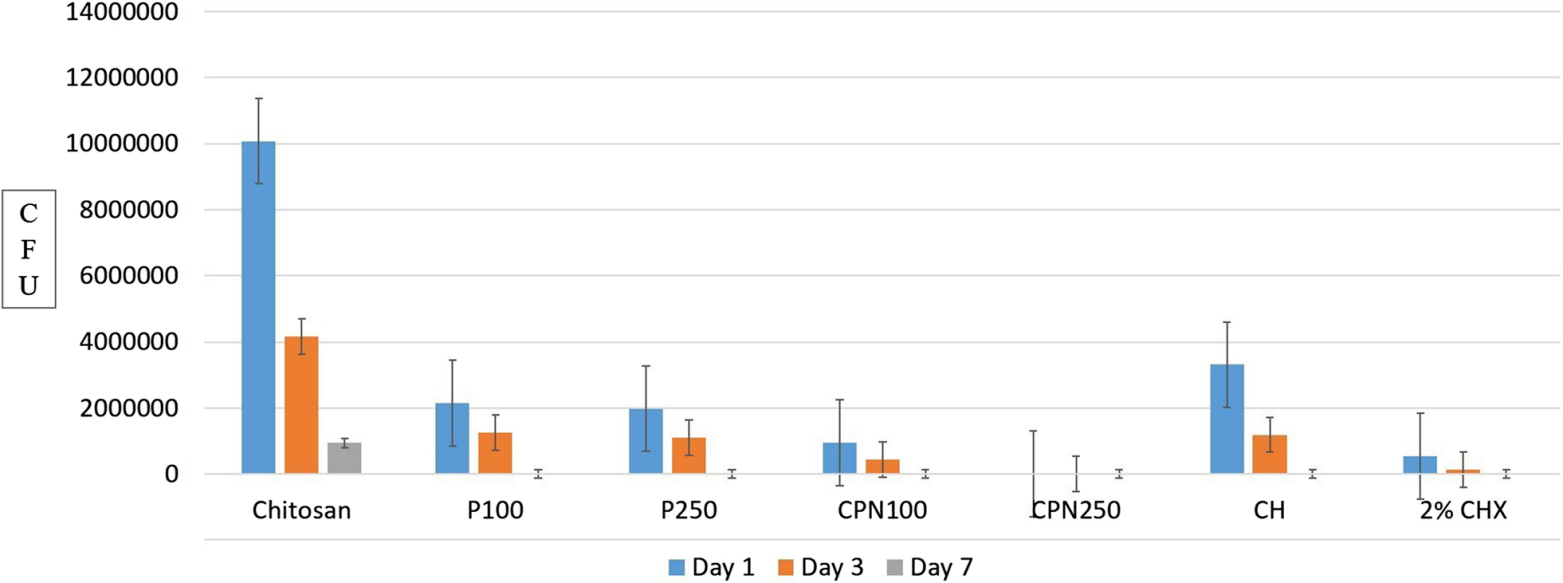
Comparison of CFUs between experimental groups on day one, three and seven at 200 µm

**Fig. 2:**
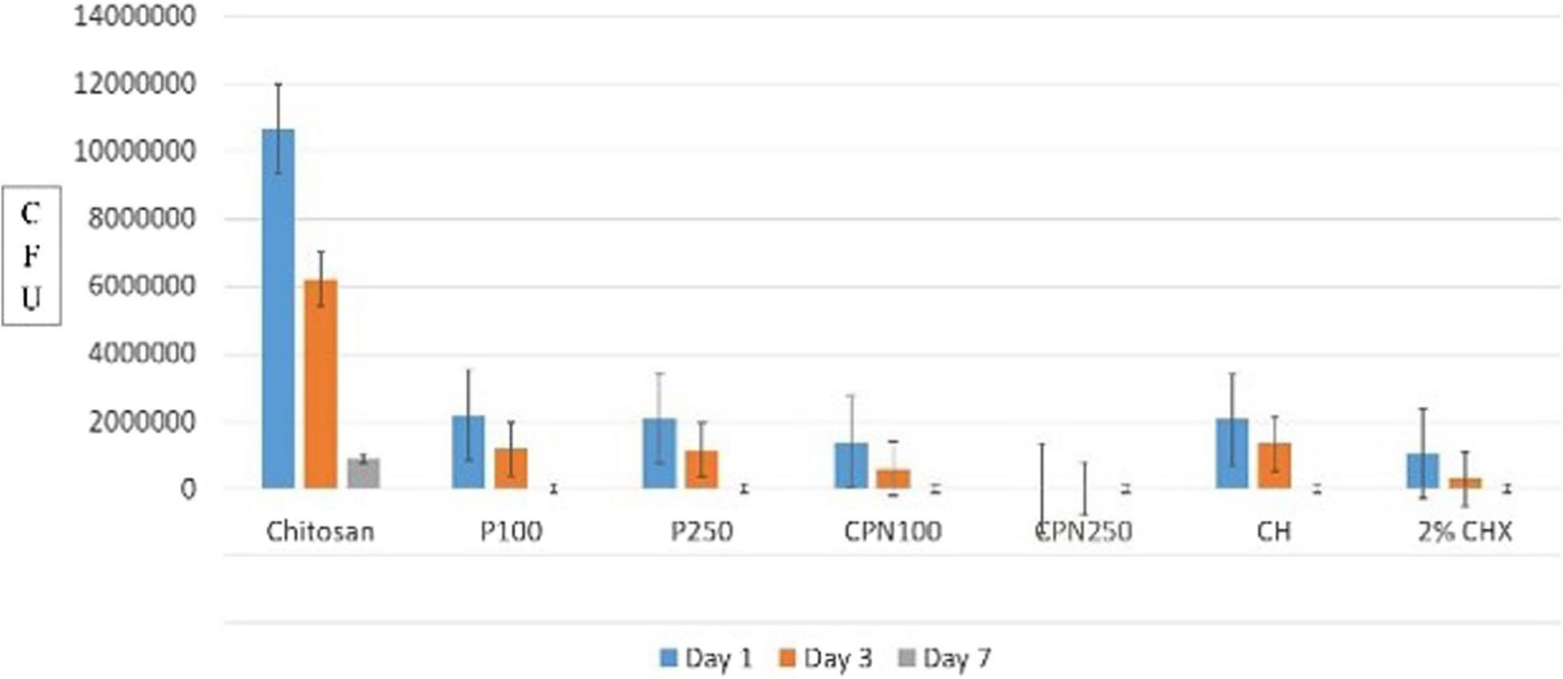
Comparison of CFUs between experimental groups on day one, three and seven at 400 µm

Comparison in between experimental groups on day one, three and seven at 200 µm and 400 µm are shown in Figs. 1and 2 to appreciate the reduction in CFUs.

Among all three time intervals CPN250 and CPN100 were most effective at day seven when compared to day one and three.

**Fig. 3:**
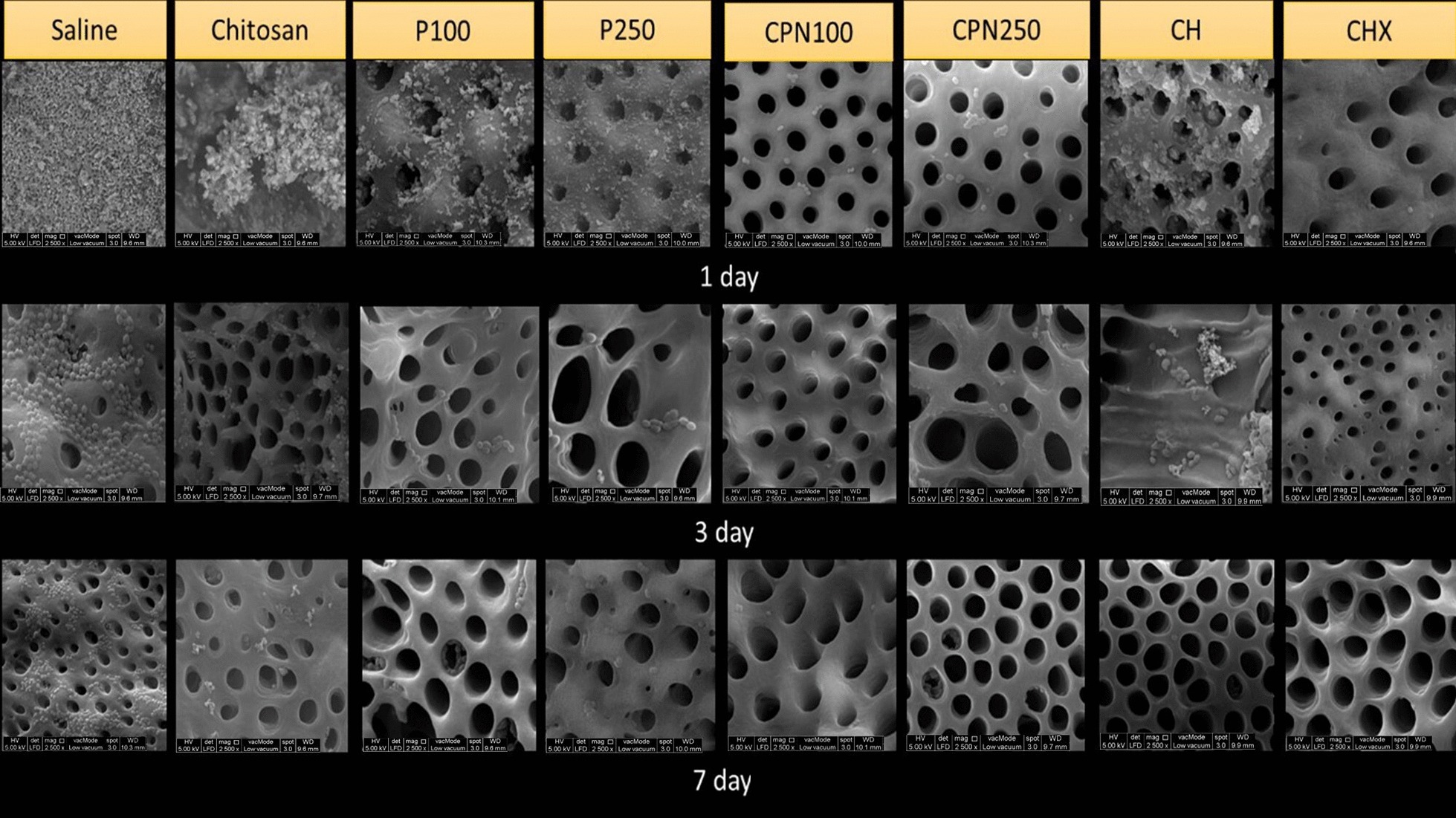
SEM images of all groups showing reduction in *E faecalis* except saline group showing large amounts of *E. faecalis*. On day one, three and seven saline group showed the highest *E. faecalis* coverage of 67–100% on SEM images of the canal wall. On day one, CPN250 and 2% CHX showed the least *E. faecalis* coverage of 5–33% while CH showed 34–66%. On day three CPN250, 2% CHX and CPN100 showed the least *E. faecalis* coverage of less than 5% while CH showed 5–33%. On day seven, CPN250, CPN100, 2%CHX and CH showed less than 5% of *E. faecalis* coverage

SEM images verified the presence of thick biofilm of residual *E. faecalis* bacteria on the root canal dentin when treated with saline. On day one, three and seven, saline group showed the highest residual *E. faecalis* coverage of 67–100% on the root canal dentin. On day one, CPN250 and 2% CHX showed the least *E. faecalis* coverage of 5–33% while CPN100, CH, P250 and P100 showed 34–66% coverage. On day three, CPN250, 2% CHX and CPN100 showed the least *E. faecalis* coverage of less than 5% while CH showed 5- 33%. On day seven, CPN250, CPN100, 2%CHX and CH showed less than 5% of *E. faecalis* coverage on the root canal dentin (Fig. 3).

**Fig. 4:**
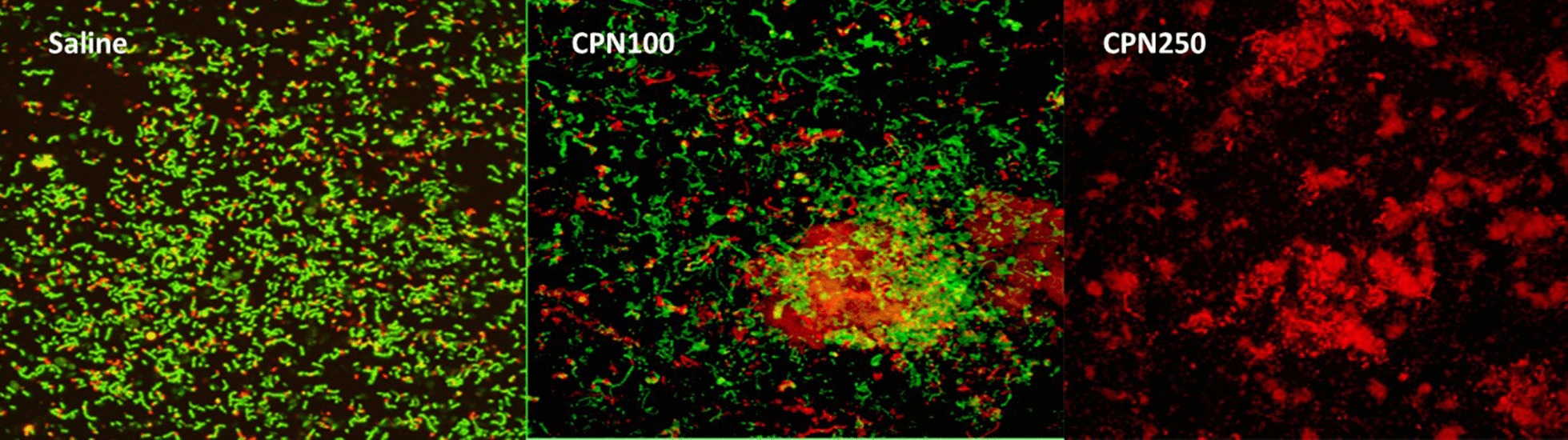
CLSM of *E. faecalis* infected dentinal blocks treated by saline (control), CPN100 and CPN250 after viability staining. CLSM image depicting green fluorescent staining indicating live bacteria in saline group, mix of green and red fluorescent staining indicating live bacteria and dead bacteria in CPN100 group and complete red fluorescent staining indicating all dead bacteria in CPN250

CLSM images also showed the amount of dead cells in dentin was highest with CPN 250 (almost 100%) compared to CPN100 (> 40%) and saline (all live cells) (Fig. 4).

### Antibacterial effect of CPN as an intracanal medicament against E. faecalis isolates from clinical samples

**Fig. 5:**
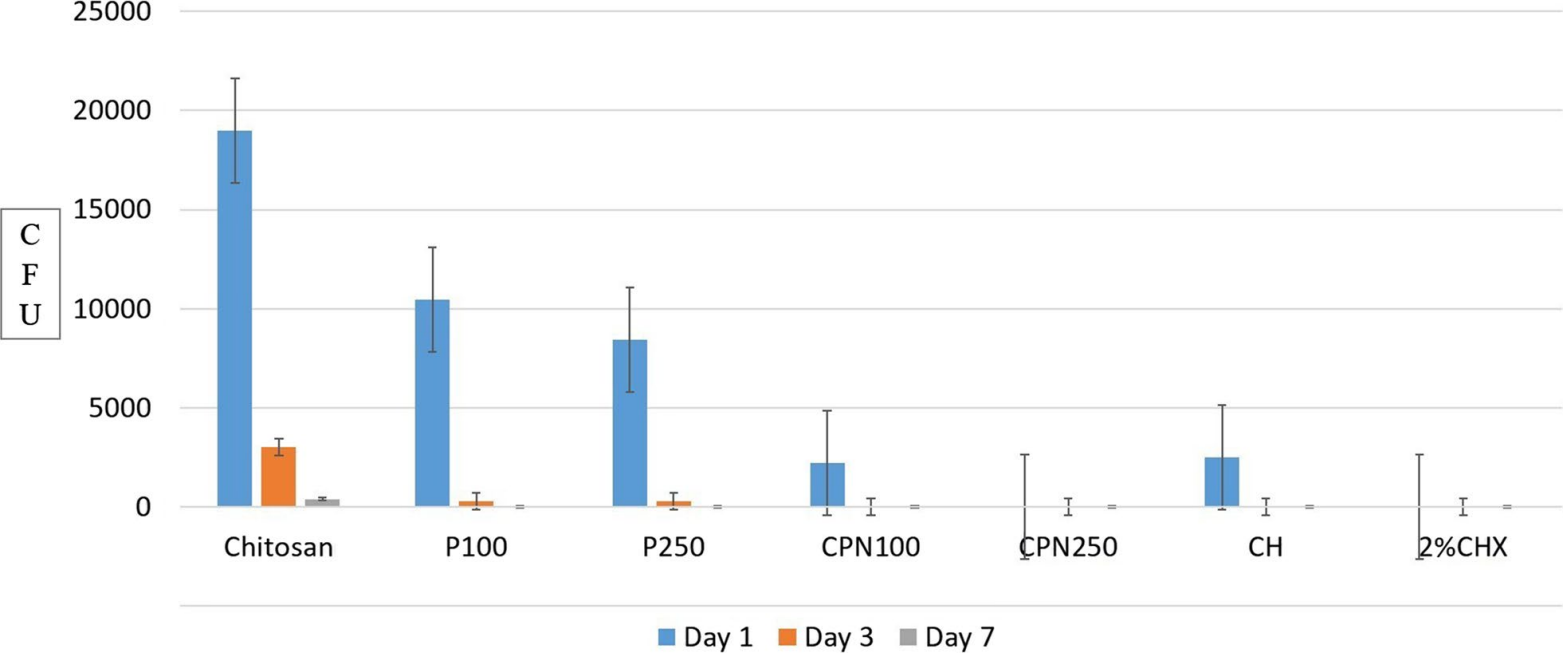
Comparison of CFUs between CPN and other intracanal medicaments on day one, three and seven against *E. faecalis* isolates from patients with failed root canal treatment

Reduction in the number of CFUs was statistically significant in all groups compared to the control group (p < 0.05). On day one, three and seven CPN250 and 2% CHX showed no growth of *E. faecalis* while CPN100, CH, P100 and P250 showed complete eradication after 3 and 5 days (Fig. 5).

## Discussion

The eradication of bacteria by endodontic treatment from the root canal has been reported as difficult mainly due to the root canal complexity and biofilm formation (6). The success of endodontic treatment depends on the chemomechanical disinfection that eliminates the vital or necrotic pulp tissue, kills microorganisms in the root canal system and disrupts microbial biofilm. This eliminates the etiological factors responsible for endodontic infection. Therefore, root canal instrumentation is always accompanied with copious irrigation to achieve chemical, mechanical and biological effects [34]. Furthermore, the use of a biocompatible intracanal medicament having optimal antimicrobial effectiveness in-between appointments may reduce or eradicate bacteria in the root canal and thereby significantly increasing the successful endodontic outcome [35].

The literature has shown that *E. faecalis* has been one of the most prevalent microorganisms ranging between 24 to 77% isolated from failed root canal cases [7–9]. Moreover, it can penetrate deep into dentinal tubules and adheres to host cells or abiotic surfaces leading to biofilm formation [15], making the disinfection very challenging. Due to this, various intracanal medicaments may not be effective against these micro-organisms. Therefore, in the present study, effectiveness of various intracanal medicaments was evaluated against the 21 days’ mature *E. faecalis* biofilm at three different time intervals because it has been shown that mature *E. faecalis* biofilms in dentin canals at 21 days are more resistant to disinfecting solutions than young biofilms [36]. Additionally, time-dependent antimicrobial effect can be useful in clinical practice to efficiently disinfect the root canal system [37, 38].

In the present study, CPN250 showed significant reduction of colony forming units compared to all other groups, however, on day 7 at 200 μm CPN100 and 2% CHX showed similar effect as CPN250, while at 400 μm CPN100, CH and 2% CHX showed similar effect as CPN250. This can be supported by the fact that reduction in the particle size of CPN 250 allows better penetration in to the dentinal tubules and enhances its efficacy [27, 39]. Del Carpio-Perochena *el al.* also found that incorporating nanoparticles could potentially be beneficial when using interappointment intracanal medications because of their ability to kill bacteria in short- and long-term exposure [40]. Furthermore, factors such as zeta potential, poly dispersity index, encapsulation of nanoparticles and rate of release of the active ingredients attribute to the antibacterial effectiveness. Zeta potential is referred to as surface electrostatic potential that strongly affects the stability of nanoparticles. Typically, stabilised nanoparticles should have zeta potential of ± 30 mV [41–43]. In the present study, CPN had high value (45.2 mV) of the zeta potential that allowed a stable and dispersed suspension which prevented the occurrence of aggregation of the nanoparticles in a short period of time. Polydispersity index is an indicator of the size distribution of nanoparticles. Polydispersity index of CPN in this study was found to be 0.225 ± 0.011 signifying a low size profile and homogenous distribution. A polydispersity index that is equal to one signifies a solution having a broad and variable nanoparticle size distribution [44]. In the present study, the encapsulation efficiency of CPN was found to be 88% that represents the drug carrying capacity of nanoparticles.

The concentrations chosen for this research such as CPN 100 and CPN 250 correspond to the minimum inhibitory concentrations and minimum bactericidal concentrations of propolis used in other similar studies [29, 45, 46]. Additionally, Bueno-Silva et al*.* [47] found the minimal inhibitory concentrations of propolis varying from 15.6 to 125 μg/ml and bactericidal concentrations varying from 31.2 to 500 μg/ml. Furthermore, concentration of propolis influences the effectiveness of CPN in reducing *E faecalis* CFUs as shown in this study. This finding is consistent with the study conducted by Pimenta et al*.* [48] where 40% of Brazilian brown propolis was more effective than 20% of its concentration against *E. faecalis*. Kim et al*.* [49] conducted a study to determine the optimal concentation of Korean propolis against *Streptococcus mutans* and reported that propolis at concentrations more than 35 µg/ml has antimicrobial activity against 90% of mutans streptococci strains. Nonetheless, optimal concentration of brown propolis against *E. faecalis* is not known yet [47]. However, other factors such as type of the raw material, plant source, temperature zone, season, time and geographic location influence the composition, characteristics and biological properties of propolis [47, 50, 51].

Seidel et al*.* [52] studied antibacterial activity of propolis from different climatic zones and observed high antibacterial activity in propolis obtained from wet‐tropical rainforest‐type climate. Bueno-Silva et al*.* [47] evaluated the effect of seasons on the chemical antibacterial property and chemical composition of Brazilian red propolis. The authors observed the highest antimicrobial activity of propolis collected in between January to May month, a period characterised by a tropical climate with rains and high relative humidity. They further suggested that the season of collection influences the quantitative chemical composition of propolis thereby affecting its biological properties.

Ethanol extracts of propolis showed high antibacterial property in this study. This could be due to presence of high level of flavonoids including pinocermbrin, kaempferol and quercetin in Malaysian propolis. Similarly, Chaillou and Nazareno [53] demonstrated strong antimicrobial activity of Argentian propolis due to the presence of high content of pinocembrin, a dihydroxy flavanone in propolis. Pinocembrin, quercetin, kaempferol and other flavonoids act on the microbial membrane or cell wall site, causing functional and structural damages [21, 22, 35].

Though, P100 and P250 in this study showed antibacterial effect but it was not as effective as CPN that can be explained due to their poor penetrability in the dentinal tubule but when prepared as nanoparticles it enhances drug stability, treatment efficacy and penetration power compared to a pure drug solution [39, 40].

In this study, 2% CHX gel showed higher effectiveness than other groups except CPN250 against *E.* faecalis at dentinal tubule depths of 200 and 400 µm on day one, three and seven. This is in accordance with studies conducted by Kandaswamy et al*.* [35], Neelkantan et al*.* [37] and Gomes et al*.* [54] where 2% CHX has been reported to be more effective than CH against *E. faecalis.* 2% of CHX gel is bactericidal and remains in contact with dentinal tubules showing property of substantivity which inhibits re-infection for a duration of at least 12 weeks [55, 56].

CH showed less effectiveness than CPN and 2% CHX on day one and three at both depths however, it was as effective as CPN and 2% CHX on day seven. CH releases hydroxyl ions resulting in a highly alkaline environment that damages the microbial cytoplasmic membrane, inhibits enzyme activity and disrupts the cellular metabolism of microorganisms [57]. These results are in accordance with other studies where CH was found to be less effective than CHX [35, 54] and propolis [58] against *E. faecalis*.

Evans et al*.* [59] have demonstrated various mechanisms involved in the resistance of *E. faecalis* to calcium hydroxide such as proton pump activity of *E. faecalis* that offers resistance to high pH of Calcium hydroxide. In this study, chitosan alone was not effective against *E. faecalis* however, as a carrier for CPN it showed the best results. Chitosan is a natural cationic polysaccharide derived by N-deacetylation of chitin exhibiting adhesiveness, biocompatibility and biodegradability [39, 40] therefore can be used in various endodontic applications [60].

In this study the extracted tooth model developed by Haapasalo & Orstavik was modified to include natural human teeth as specimens, thereby provided a better simulation to the clinical settings to assess the efficacy of intracanal medicaments in the disinfection of dentinal tubules [61]. Mid root dentin blocks of the root canal were maintained to a standard of 6.0 mm in height and 0.9 mm in diameter to ensure placing a constant amount of bacteria during inoculation, and intracanal medicaments. The samples were tested at two depths of dentinal tubules, 200 µm and 400 µm, because intracanal medicament such as calcium hydroxide is known to penetrate only upto 200–300 µm [62].

Quantitative analysis of bacteria in the dentine tubules was done to define a log reduction in CFUs in infected dentine before and after the application of intracanal medicaments. CFUs methodology has been widely used for microbiological analysis of bacteria inside the dentinal tubules. Although it was able to provide a reading of the bacterial colony that had invaded the dentinal tubules, it was unable to analyse spatial distribution and viability of the bacteria. In the present study *E. faecalis* mono-species bioflm has been used which is in accordance with Swimberghe et al*.* [63] who presented an outline of laboratory root canal biofilm model systems and critically appraised the factors that constitute these models. The authors observed that most of the included studies (86%) used mono-species biofilm. *E. faecalis* was the most frequently used test species in 92% of the mono-species studies and 79% of all studies. Human dentine was the most frequently used substratum in 88% of the studies with different incubation time ranging from one to seventy days. Furthermore, bacterial culturing was found to be the most common quantification method used followed by microscopy techniques.

This research project is one of its kind as CPN has never been tested before. Furthermore, in this research, along with the extracted teeth, *E. faecalis* isolates were obtained from patients with failed root canal treatment to evaluate the effectiveness of CPN.

For further analysis, this study has used SEM for all groups and CLSM only for saline, CPN100, CPN250 as intracanal medicament. This is the first project in which CLSM was performed to evaluate the antibacterial effectiveness of CPN and saline. However, CLSM analysis for the remaining groups can be conducted in future.

### Future recommendations

The antimicrobial effectiveness of CPN250 as an intracanal medicament should be evaluated against polymicrobial biofilm and its disruption in future studies. To further strengthen the evidence, future animal studies and clinical trials are warranted. The antimicrobial effect of CPN250 can also be compared with other nanoparticles such as silver and gold in future studies.

Antimicrobial activity of Malaysian propolis has not been studied in depth therefore, further research is required to understand and elucidate its mechanism of action, especially at the cellular level.

## Conclusions

CPN250 was the most effective in reducing *E. faecalis* CFUs on day one and day three at both 200 and 400 μm dentinal tubule depths and at day seven CPN250 was equally effective as CPN100 and 2% CHX. Therefore, CPN250 can be proposed as a potential intra-canal medicament to be used in future.

CPN100 was more effective in reducing *E. faecalis* CFUs than saline, chitosan, P100 and P250, on day one, three and seven at both depths and at day seven, CPN100 was equally effective as CPN 250, CH and 2% CHX.

CPN250 and CPN100 as intracanal medicaments were the most effective on day seven in reducing *E. faecalis* CFUs when compared to day one and day three.

Additionally, CPN250 and CPN100 were found to be effective as intracanal medicaments in reducing *E. faecalis* isolates obtained from patients with failed root canal treatment.

## Data Availability

The data analyzed during this present study are available from corresponding author on request.

## Abbreviations

CFUs: Colony forming units
CH: Calcium hydroxide
CHX: Chlorhexidine
CLSM: Confocal laser scanning microscopy
CPN100: Chitosan-propolis nanoparticle 100 µg/ml
CPN250: Chitosan-propolis nanoparticle 250 µg/ml
*E. faecalis*: *Enterococcus faecalis*
P100: Propolis 100 µg/ml
P250: Propolis 250 µg/ml
SEM: Scanning electron microscope
w/v: Weight per volume
TSB: Tryptic soy broth
kV: Kilo volt
v/v: Volume per volume
